# Supplementary material for: Path-dependent connectivity, not modularity, consistently predicts controllability of structural brain networks
Source: Netw Neurosci. 2020 Nov 1;4(4):1091–121. doi: 10.1162/netn_a_00157 (PMC7655114; doi:10.1162/netn_a_00157)
Supplement: Supplementary file 1 [file netn-04-1091-s001.pdf]

Patankar, S. P., Kim, J. Z., Pasqualetti, F. & Bassett, D. S. (2020). Supporting information for "Pathdependent connectivity, not modularity, consistently predicts controllability of structural brain networks." *Network Neuroscience*, *4*(4), 1091-1121. https://doi.org/10.1162/netn_a_00157

Supplement: Path-dependent connectivity, not modularity, consistently predicts controllability of structural brain networks

Shubhankar P. Patankar[a], Jason Z. Kim[a], Fabio Pasqualetti[b], and Danielle S. Bassett[a,c,d,e,f,g,h]

[a]Department of Bioengineering, School of Engineering and Applied Science, University of Pennsylvania, Philadelphia, PA 19104

[b]Department of Mechanical Engineering, University of California, Riverside, CA 92521, USA

[c]Department of Neuroscience, Perelman School of Medicine, University of Pennsylvania, Philadelphia, PA 19104

[d]Department of Electrical and Systems Engineering, School of Engineering and Applied Science, University of Pennsylvania, Philadelphia, PA 19104

[e]Department of Neurology, Perelman School of Medicine, University of Pennsylvania, Philadelphia, PA 19104

[f]Department of Physics and Astronomy, College of Arts and Sciences, University of Pennsylvania, Philadelphia, PA 19104

[g]Department of Psychiatry, Perelman School of Medicine, University of Pennsylvania, Philadelphia, PA 19104

[h]Santa Fe Institute, Santa Fe, NM 87501

1 Application of Linear Network Control Theory to Neural Systems

Modeling techniques in network neuroscience span multiple dimensions, ranging from data representations to first-principles theory, from biophysical to functional realism, and from elementary to coarse-grained approximations [Bassett et al., 2018]. Descriptive statistics such as node strength, small-worldness, centrality and modularity are commonly used in network neuroscience, and offer valuable insight into the function of neural systems without attempting to accurately model neural dynamics. The linear model used in the paper is not intended to be a biophysically realistic depiction of brain network function, but rather to be a theoretical model with a few biologically plausible constraints. These constraints include the connections between brain regions as measured by streamline counts, the stability of brain dynamics, and the ability to exert endogenous or exogenous influence on them. Early results obtained using statistics derived from the application of linear control theory to structural brain networks are promising.

Preliminary work in neural systems indicates that average controllability, as defined in the context of a linearized model of network dynamics, is able to predict transitions in and out of bursting regimes in neural ensembles [Wiles et al., 2017]. Predictions from linear controllability are consistent with simulations of neuronal networks with nonlinear dynamics [Whalen et al., 2015, Muldoon et al., 2016]. Yan *et al.* use linear network control theory with the *C. elegans* connectome to make predictions regarding neurons that may be implicated in locomotion, and then experimentally validate their findings [Yan et al., 2017]. Beynel *et al.* find that modal controllability tracks empirically observed brain state transitions measured using fMRI during a working memory task [Beynel et al., 2019]. Neurotransmitter groups can be separated using average and modal controllability into those that facilitate cognition and those that do not [Shine et al., 2019].

Recent work has demonstrated a link between network controllability and executive function [Cornblath et al., 2020, Cui et al., 2020]. Cornblath *et al.* find associations between average controllability and individual differences in activation during an n-back working memory task [Cornblath et al., 2020]. Cui *et al.* observe that the energetic requirements to activate the fronto-parietal system decline with age in normative neurodevelopment [Cui et al., 2020]. In a complementary study, it has been noted that the capacity of individual brain regions to alter whole brain dynamics changes with age [Tang et al., 2017]. Network control theory has also been applied to invasive neuromodulation data to predict response to electrical stimulation in theory [Muldoon et al., 2016], as well as in practice [Stiso et al., 2019, Khambhati et al., 2019]. A number of these promising studies relate predictions from linear network control theory to independently measured neurobiological variables.

Yang *et al.* use a linear state space model on top of a network to perform one-step-ahead prediction with electrocorticogram (ECoG) data [Yang et al., 2019a]. They find that adding a general nonlinearity through Gaussian radial basis functions does not outperform the linear model. As a result, a generally nonlinear model is not guaranteed to outperform a linear one, unless the specific functional form of the nonlinearity in dynamics is known. Therefore, a simple linear model is a useful starting point to probe network dynamics. The identification of a specific nonlinearity is outside the scope of the current work, given that we seek to characterize how mesoscale structure affects the measures derived from linear network control theory in the context of structural brain networks.

As noted in the main manuscript, the linear framework can be extended to incorporate more

complex features of neural dynamics [Li et al., 2017, Yang et al., 2019b, Zañudo et al., 2017]. Prior work has extended linear models to include piecewise linearities which get closer to modeling non-linear dynamics. For instance, Yang *et al.* approximate non-linear dynamics of medically induced coma using a linear two-compartment model with local Taylor series expansions about shifting fixed points [Yang et al., 2019b]. An LTI model can also be extended to incorporate feedback in order to account for recurrence.

2 Motivation for Target States for Minimum Control Energy

When working with minimum control energy, we perform N distinct state transitions corresponding to each node in turn, where the initial state vector is the zero vector, and the target states are N one-hot vectors intended to simulate the activation of individual brain regions. For each of the 234 brain regions, we separately compute the energy required to change the state of node i from 0 to 1, while keeping other brain regions inactive (from 0 to 0), for $i = 1, \cdots, N$.

This choice of final states creates an upper bound on the energy required to perform a state transition from the zero vector to an arbitrary non-negative target vector $\boldsymbol{x}^*$. To demonstrate this point, let E_i represent the energy required to bring our system from an initial state of $\boldsymbol{x}(0) = \mathbf{0}$ to a final state $\boldsymbol{e}_i$ that is a one-hot vector with a 1 in the i-th entry, and 0s elsewhere. The energy metric we compute is precisely E_i for $i = 1, \cdots, N$. Suppose we wanted to reach another target state, $\boldsymbol{x}^*$, that we can write using the vectors $\boldsymbol{e}_i$ as basis vectors

$$\boldsymbol{x}^* = \sum_{i=1}^{N} x_i^* \boldsymbol{e}_i,$$

where $x_i^* \geq 0$ is the i-th element of $\boldsymbol{x}^*$. The energy required to perform this state transition to $\boldsymbol{x}^*$ is given by

$$\begin{aligned} E_{\boldsymbol{x}^*} &= \boldsymbol{x}^{*\top} W c^{-1} \boldsymbol{x}^* \\ &= \sum_{i=1}^{N} x_i^{*2} \boldsymbol{e}_i^\top W_c^{-1} \boldsymbol{e}_i + \sum_{i=1}^{N} \sum_{j \neq i} x_i^* x_j^* \boldsymbol{e}_i^\top W_c^{-1} \boldsymbol{e}_j. \end{aligned}$$

where W_c is the controllability Gramian. In this expression, we observe that in the first summation term, the expression $\boldsymbol{e}_i^\top W_c^{-1} \boldsymbol{e}_i$ is precisely the energy we compute, E_i, such that

$$E_{\boldsymbol{x}^*} = \sum_{i=1}^{N} x_i^{*2} E_i + \sum_{i=1}^{N} \sum_{j \neq i} x_i^* x_j^* \boldsymbol{e}_i^\top W_c^{-1} \boldsymbol{e}_j. \tag{1}$$

We see that the first summation term is the independent energies E_i, while the second summation term encodes the energetic cost due to interactions. Next, we demonstrate that all of the cross terms become negative when $x_i, x_j > 0$.

Let us consider what happens for a normalized symmetric matrix (largest eigenvalue of A has magnitude $\lambda = 1$) to the cross term at the limit of T approaching infinity. Then the controllability Gramian can be written as

$$W_c = I + A^2 + A^4 + \cdots = \lim_{n \to \infty} (I - A^{2n})(I - A^2)^{-1},$$

as the Gramian becomes a geometric series with respect to matrix A, whose convergence is a function of the matrix's eigenvalues. As such, according to the spectral mapping theorem, we can write the convergence of the series with respect to the matrix A. If we invert this Gramian, we obtain

$$W_c^{-1} = \lim_{n \to \infty} (I - A^{2n})^{-1}(I - A^2).$$

Based on our normalization scheme, most of the eigenvalues of A have magnitude less than 1. Because taking powers of a matrix A corresponds to taking powers of its eigenvalues, the value of A^{2n} at the limit of infinite n turns these eigenvalues to zero:

$$\lim_{n\to\infty} \lambda_{\text{not max}}^{2n} = 0,$$

such that the convergence of these modes in the Gramian inverse becomes

$$\chi_i^{-1} = \lim_{n\to\infty} \frac{1-\lambda_i^2}{1-\lambda_i^{2n}} = 1 - \lambda_i^2,$$

where χ_i is the i-th eigenvalue of the Gramian. As for the largest eigenvalue of A that has magnitude 1, the convergence of the geometric series goes to 0 based on L'Hópital's rule:

$$\lim_{n\to\infty} \frac{1-\lambda_{\text{max}}^2}{1-\lambda_{\text{max}}^{2n}} = \lim_{n\to\infty} \frac{-2\lambda_{\text{max}}}{-2n\lambda_{\text{max}}^{2n-1}} = \lim_{n\to\infty} \frac{1}{n} = 0 = 1 - \lambda_{\text{max}}^2,$$

for $\lambda_{\text{max}} = 1$. Hence, we observe that all of the eigenvalues of the inverse of the Gramian can be written as $1-\lambda_i^2$, and because the matrix A, the Gramian W_c, and the Gramian inverse W_c^{-1} all preserve the same eigenspace, we use the spectral mapping theorem to write

$$\lim_{n\to\infty} W_c^{-1} = I - A^2.$$

Because A is non-negative, all of the entries of A^2 are also non-negative. Hence, all of the non-diagonal entries of W_c^{-1} are negative, such that all of the cross-terms in equation 1 are negative. Hence, the energy E_i to drive the system from an initial zero vector to a one-hot target vector $\boldsymbol{e}_i$ provides an upper bound on the energy required to perform state transitions to arbitrary non-negative target states $\boldsymbol{x}^*$.

3 Robustness of Results to the Choice of Time Horizon

Note: all scatter plots in the Supplement reflect results of partial Spearman correlations after correcting for node strength.

In the main manuscript we present minimum control energy and average controllability results for a time horizon choice of $T = 4$. Recall that we find that node-level measures of modularity such as participation coefficient and intra-module strength Z-score are unable to consistently predict measures of network controllability such as minimum controllability, average controllability, and modal controllability when accounting for the effect of node strength. On the other hand, weighted subgraph centrality is a robust node-level predictor of measures of network controllability even when node strength is included as a covariate. Here, we demonstrate that the node strength corrected relationship between weighted subgraph centrality and network controllability remains robust for a wide-range of values of the time horizon T.

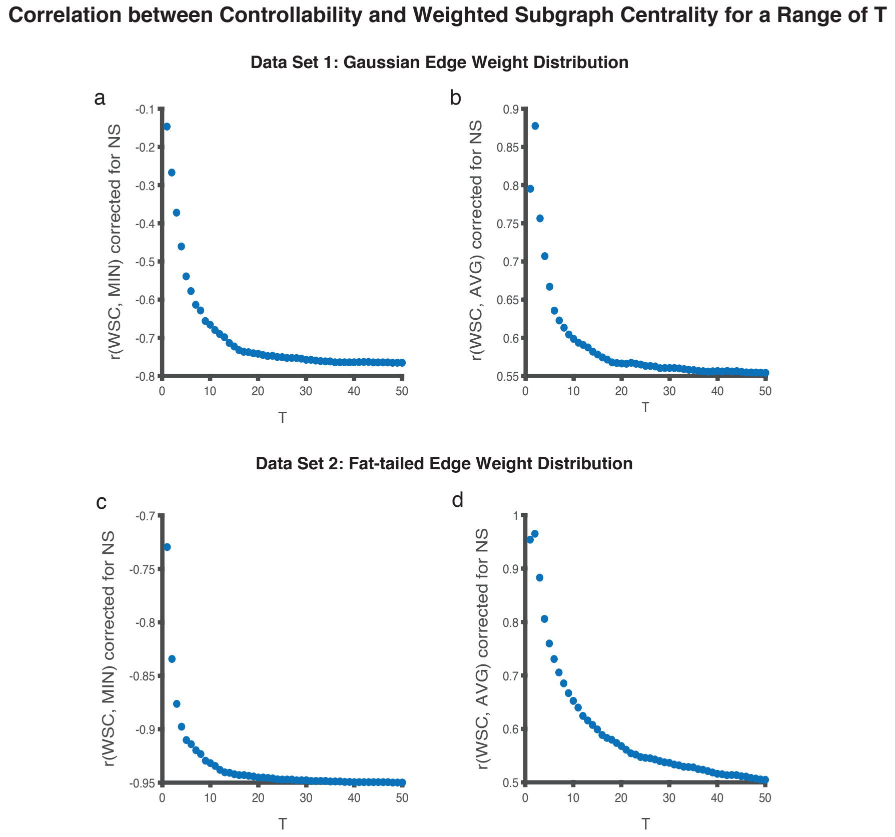

Figure 1: **Spearman correlation coefficients between weighted subgraph centrality and minimum control energy or average controllability corrected for node strength remain high for $T = [1, 50]$.** **(a-d)** Weighted subgraph centrality corrected for node strength predicts network controllability in a statistically significant manner for a wide range of values of the time horizon T. Correlations between weighted subgraph centrality and network controllability generalize across structural brain network data sets with very distinct edge weight distributions. **(a, b)** Data Set 1 is comprised of networks with normally distributed edge weights, **(c, d)** whereas Data Set 2 is comprised of networks with a fat-tailed edge weight distribution.

4 Alternate Choice for Edge Weight Distribution Prior

Next we demonstrate the robustness of our analyses to an alternative choice of edge weight distribution prior. For Data Set 2, network edges approximate a fat-tailed distribution. In the main manuscript, we infer communities in these networks with the weighted stochastic block model using the log-normal distribution as the prior choice of edge weight distribution. Here we repeat our analyses with the normal distribution as the choice of edge weight distribution prior. Consistent with the results presented in the main manuscript, we perform minimum control energy and average controllability computations with $T = 4$. All correlations are performed with node strength included as a covariate.

We confirm that the lack of a relationship between measures of modularity and measures of controllability persists regardless of the edge weight distribution prior pre-supposed when inferring communities with the WSBM. We do not re-produce results for correlations between weighted subgraph centrality and network controllability, since the values of weighted subgraph centrality are not determined by a network's community structure, and as such are not affected by how the WSBM is applied to the data.

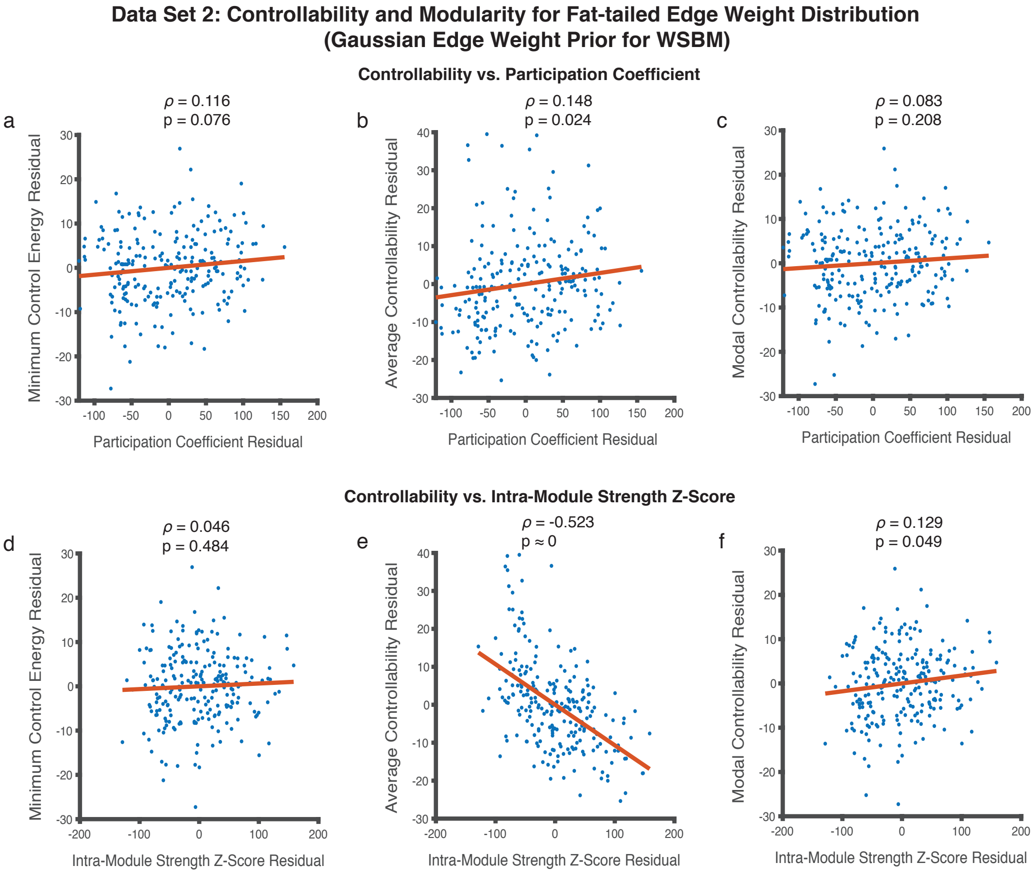

Figure 2: **Data Set 2, communities inferred using a Gaussian edge weight distribution prior.** (**a-c**) Participation coefficient is not related to minimum control energy ($\rho = 0.116$, $p = 0.076$), or to modal controllability ($\rho = 0.083$, $p = 0.208$). It relates positively with average controllability ($\rho = 0.148$, $p = 0.024$). (**d-f**) Intra-module strength Z-score does not relate with minimum control energy ($\rho = 0.046$, $p = 0.484$). It relates negatively with average controllability ($\rho = -0.523$, $p \approx 0$), and positively with modal controllability ($\rho = 0.129$, $p = 0.049$). Each dot in the scatter plots represents the mean value of a controllability and modularity measure across 24 (8 subjects in triplicate) network instantiations for a single brain region.

5 Results with an Alternative Data Set

Data Set 1 and Data Set 2 are comprised of structural brain networks inferred from diffusion spectrum imaging (DSI) of eight subjects in triplicate. Data Set 1 consists of networks with estimates of mean quantitative anisotropy (QA) values between regions acting as edge weights. The distribution of network edges from this data set approximates a normal distribution. Data Set 2, on the other hand, is comprised of networks with streamline counts between regions assigned as values to edges. The distribution of edges in this data set approximates a fat-tailed distribution. We find that weighted subgraph centrality is a consistent predictor of network controllability that generalizes across these two distinct distributions of network edge weights. In order to confirm this finding, we turn to a third higher-resolution data set termed Data Set 3, which is comprised of structural brain networks inferred from ten subjects. Edges in the networks in this data set are values of streamline counts between regions corrected for the volumes of the regions. The distribution of network edge weights in this data set also approximates a fat-tailed distribution.

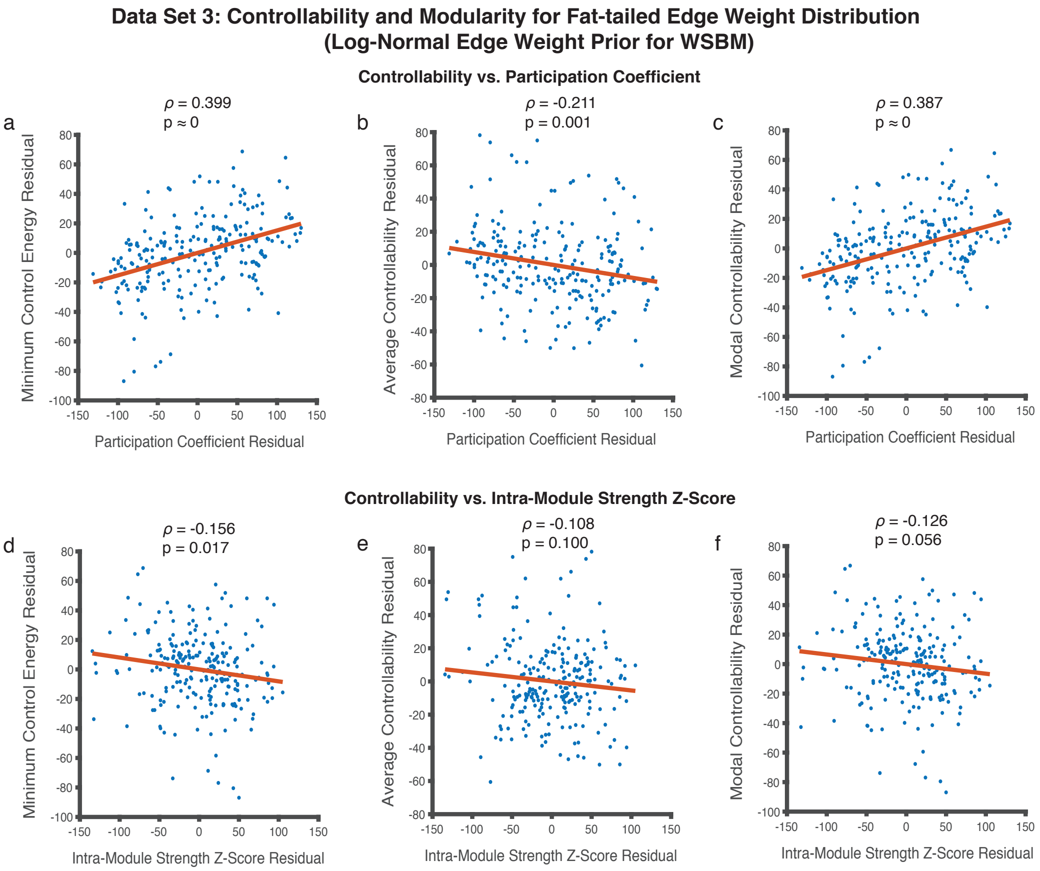

Figure 3: **Data Set 3 (higher resolution data set), communities inferred from networks with volume normalized streamline counts as edges with log-normal prior.** $T = 4$ **for minimum control energy and average controllability.** (**a-c**) Participation coefficient is positively related to minimum control energy ($\rho = 0.399$, $p \approx 0$) and modal controllability ($\rho = 0.387$, $p \approx 0$), and negatively related to average controllability ($\rho = -0.211$, $p = 0.001$). (**d-f**) Intra-module strength Z-score is negatively related with minimum control energy ($\rho = -0.156$, $p = 0.017$). It is not related with average controllability ($\rho = -0.108$, $p = 0.100$) or modal controllability ($\rho = -0.126$, $p = 0.056$). Each dot in the scatter plots represents the mean value of a controllability and modularity measure across 10 (10 subjects) network instantiations for a single brain region.

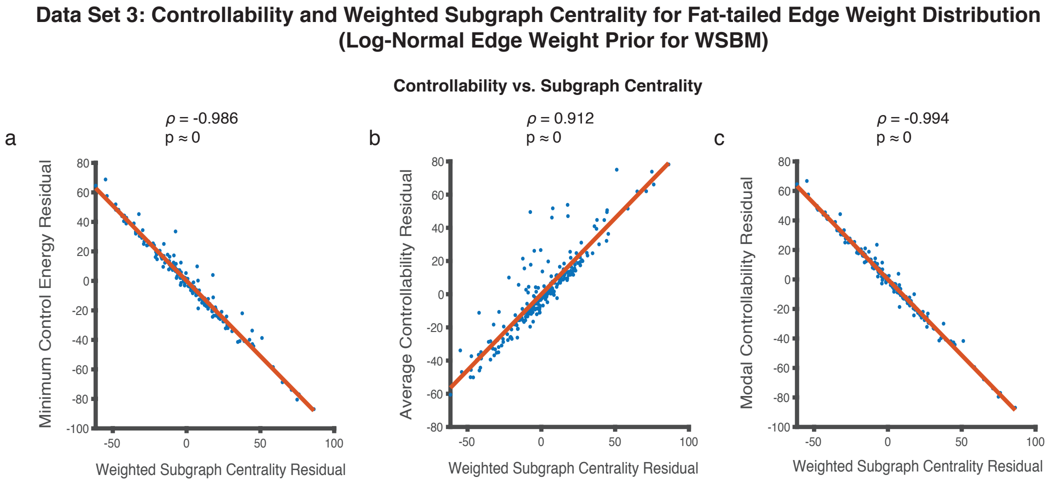

Figure 4: **Data Set 3 (higher resolution data set), communities inferred from networks with volume normalized streamline counts as edges with log-normal prior.** $T = 4$ **for minimum control energy and average controllability.** (**a-c**) Weighted subgraph centrality relates negatively to minimum control energy ($\rho = -0.986$, $p \approx 0$), and to modal controllability ($\rho = -0.994$, $p \approx 0$). It relates positively with average controllability ($\rho = 0.912$, $p \approx 0$). Each point in the scatter plots represents the mean value of a controllability measure and weighted subgraph centrality across 10 (8 subjects) network instantiations for a single brain region.

From Figure 4, we see that the trends in the relationships between weighted subgraph centrality and measures of network controllability are identical to those observed in Data Set 1 and Data Set 2.

6 Node Strength Compared to Weighted Subgraph Centrality

We note that node strength is a consistently strong predictor of measures of network controllability across data sets with distinct edge weight distributions. For instance, in Data Set 2 which is comprised of 24 structural brain networks (8 subjects scanned in triplicate) weighted by the streamline counts between regions, values of the Spearman rank correlation between minimum control energy, average controllability, and modal controllability are $\rho = -0.993$, $\rho = 0.984$, and $\rho = -0.993$, respectively. Additionally, these high correlation values persist in networks from the other two data sets examined in this paper. As a result, the practical utility of weighted subgraph centrality as a consistently strong predictor of network controllability is not obvious.

However, we demonstrate that controllability does not always correlate so strongly with node strength by considering the 24 networks (8 subjects across 3 scanning sessions) of streamline counts from Data Set 2. In each of these 24 networks, we compute the average controllability of each node (234 values for each of 24 networks), average them across networks (234 mean values), and perform a Spearman rank correlation against either the node strength or weighted subgraph centrality averaged across networks at multiple time horizons T (Figure 5a). We observe that at small values of T, node strength correlates strongly with average controllability. However, as the time horizon increases, this correlation decreases. We also observe that weighted subgraph centrality consistently performs better than node strength. This effect is stronger when considering the Pearson correlation (Figure 5b).

For control energy, we observe similar performance trends. Here, we use the same 24 networks, and compute the minimum control energy required to bring the system from an initial state of $\boldsymbol{x}(0) = \mathbf{0}$, to a final state that is 1 for node i, and zero elsewhere. We compute this energy for each node for each network (234 energies for 24 networks), average them across networks (234 mean energies), and perform both a Spearman (Figure 5c) and Pearson (Figure 5d) correlation with either node strength or weighted subgraph centrality across time horizons. We observe that while the magnitude of the Spearman rank correlation remains high for both node strength and weighted subgraph centrality, the magnitude of the Pearson correlation with node strength decreases at longer time horizons. Hence, for both average controllability and control energy, node strength is consistently outperformed by weighted subgraph centrality, and is often a worse predictor at longer time horizons.

Next, we demonstrate that weighted subgraph centrality is a better *linear* predictor of controllability. Specifically, we plot the 234 controllability values against node strength or weighted subgraph centrality, and find that the higher Pearson correlation with weighted subgraph centrality is driven by a stronger linear trend. In Figure 5e, we observe that average controllability increases in a quadratic manner with node strength, while this increase follows weighted subgraph centrality much more linearly in Figure 5f. The same comparison is true for minimum control energy in Figure 5g, and h. Hence, compared to node strength, we find that weighted subgraph centrality both correlates more strongly and linearly than node strength across many time horizons.

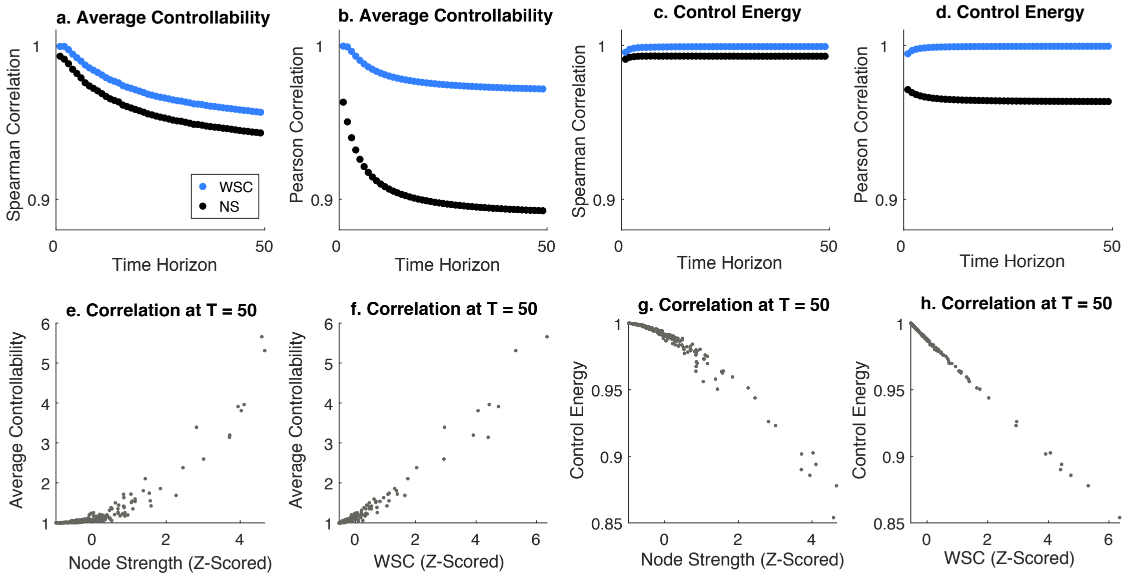

Figure 5: **Weighted subgraph centrality preserves higher linear correlation than node strength with increasing time horizon.** **(a)** The correlation coefficient between average controllability and either node strength (black) or weighted subgraph centrality (blue) across time horizons from $T = 1$ to $T = 50$ for Spearman, and **(b)** Pearson correlation. **(c)** The magnitude of the correlation coefficient between control energy and either node strength (black) or weighted subgraph centrality (blue) across time horizons from $T = 1$ to $T = 50$ for Spearman, and **(d)** Pearson correlation. **(e)** Plot of average controllability (where each point is the average controllability of each brain region averaged across 8 subjects and 3 scans) against node strength, and **(f)** weighted subgraph centrality. **(g)** Plot of control energy (where each point is the average energy to bring one brain region from a state of 0 to a state of 1, averaged across 8 subjects and 3 scans) against node strength, and **(h)** weighted subgraph centrality.

7 Effect of Asymmetry on the Relationship between Weighted Subgraph Centrality and Network Controllability

The *in vivo* imaging methods currently available to infer structural brain networks in humans are unable to resolve the directionality of edges between brain regions. In order to examine whether the relationship between weighted subgraph centrality and network controllability remains robust to asymmetry in networks, we perform analyses by first deleting half of all network edges at random in our structural brain network data sets. This process artificially induces asymmetry in the weighted network adjacency matrices that we use as system matrices in the discrete-time LTI model. We repeat salient analyses on asymmetric networks from Data Set 1, as well as Data Set 2.

We note that the trends in the relationships between weighted subgraph centrality and minimum control energy, and average controllability computed on asymmetric networks are identical to those observed with symmetric networks.

Relations between weighted subgraph centrality and modal controllability are not examined in Figure 6, since the version of modal controllability used in this paper explicitly assumes symmetric graphs.

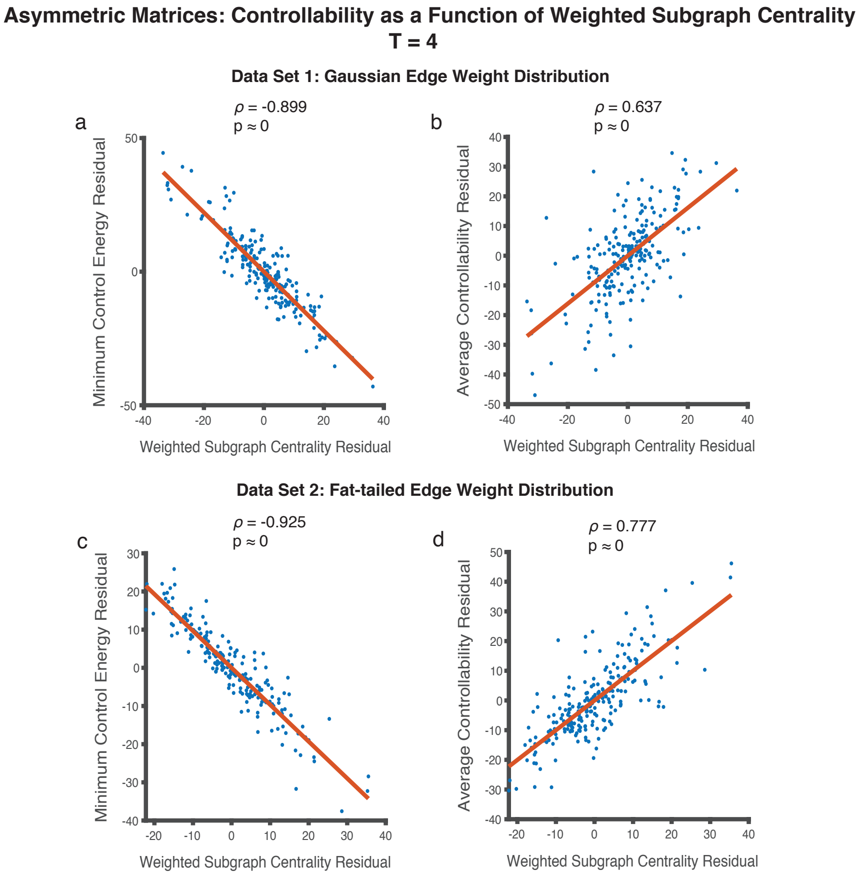

Figure 6: **Asymmetric matrices and weighted subgraph centrality.** Weighted subgraph centrality is a statistically significant predictor of minimum control energy and average controllability across the two data sets, even when the networks in the data are deliberately made asymmetric. (**a-b**) For Data Set 1, where the distribution of edge weights approximates the normal distribution, weighted subgraph centrality relates negatively with minimum control energy ($\rho = -0.899$, $p \approx 0$), and positively with average controllability ($\rho = 0.637$, $p \approx 0$). The trends remain identical for Data Set 2, where the edge weights approximate a fat-tailed distribution. Weighted subgraph centrality relates negatively with minimum control energy ($\rho = -0.925$, $p \approx 0$), and positively with average controllability ($\rho = 0.777$, $p \approx 0$).

8 Determining k

The weighted stochastic block model requires that the number of partitions k in a network be chosen *a priori* in order to infer communities. We sweep across a range of values of k in order to choose the value that maximizes the likelihood of observing a given network. For Data Set 1 and 2, at each k value we run the WSBM 10 times. This corresponds to 30 total runs for a single subject since the data is comprised of structural connectivity matrices inferred in triplicate. For Data Set 3, we run the block model a total of 25 times. Figures below plot the mean log likelihood of observing a data set given the choice of k and the prior over the edge weight distribution.

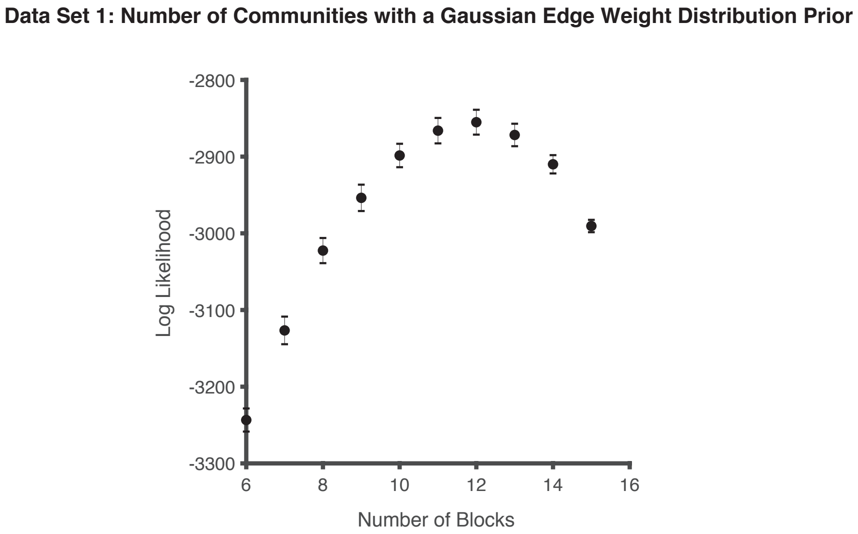

Figure 7: **Data Set 1 with Gaussian edge weight prior**. Likelihood is maximized when $k = 12$.

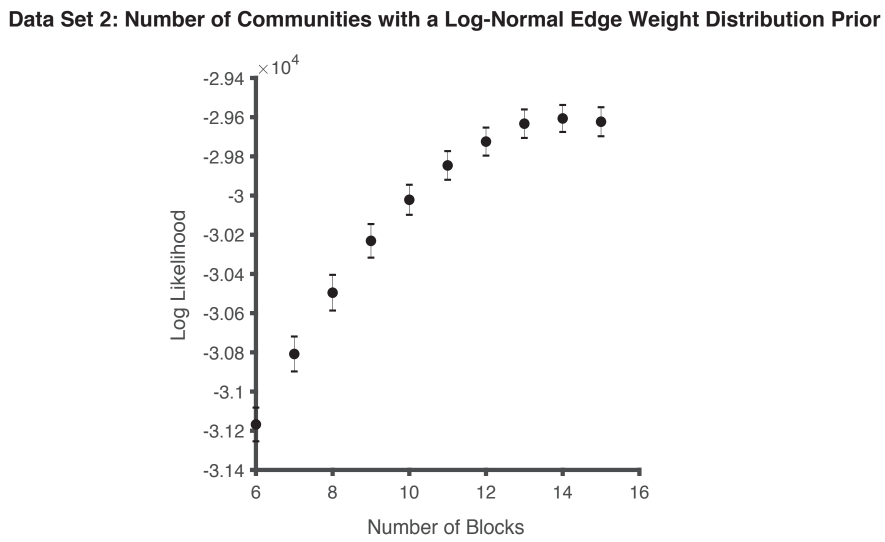

Figure 8: **Data Set 2 with log-normal edge weight prior**. Likelihood is maximized when $k = 14$.

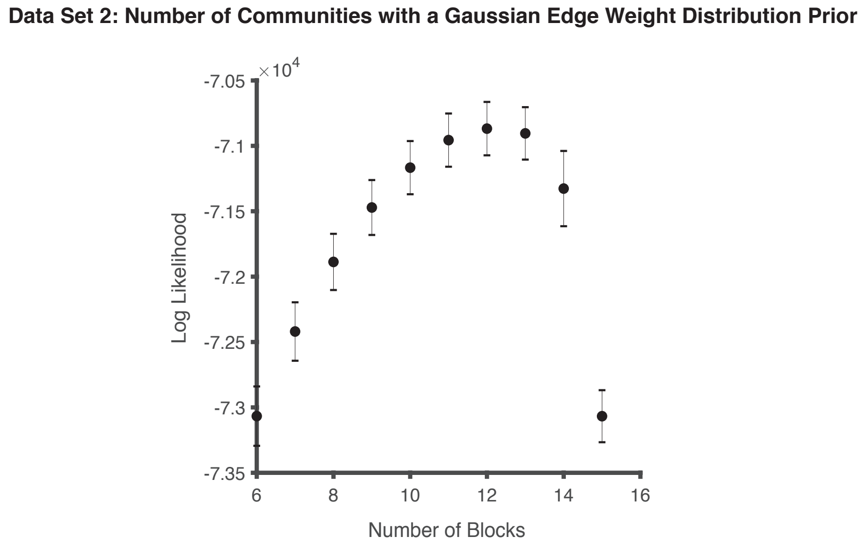

Figure 9: **Data Set 2 with Gaussian edge weight prior**. Likelihood is maximized when $k = 12$.

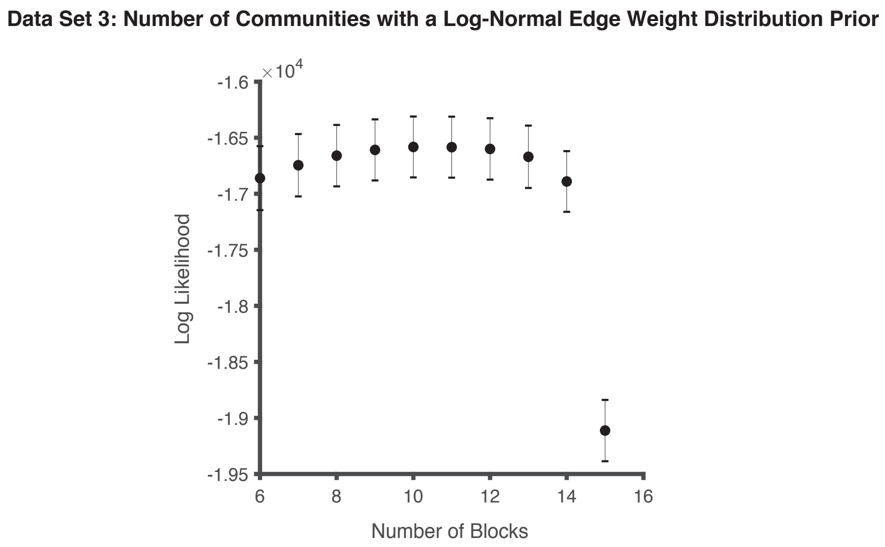

Figure 10: **Data Set 3 with log-normal edge weight prior**. Likelihood is maximized when $k = 10$.

9 Modularity Quality Q and Motifs of Network Community Structure

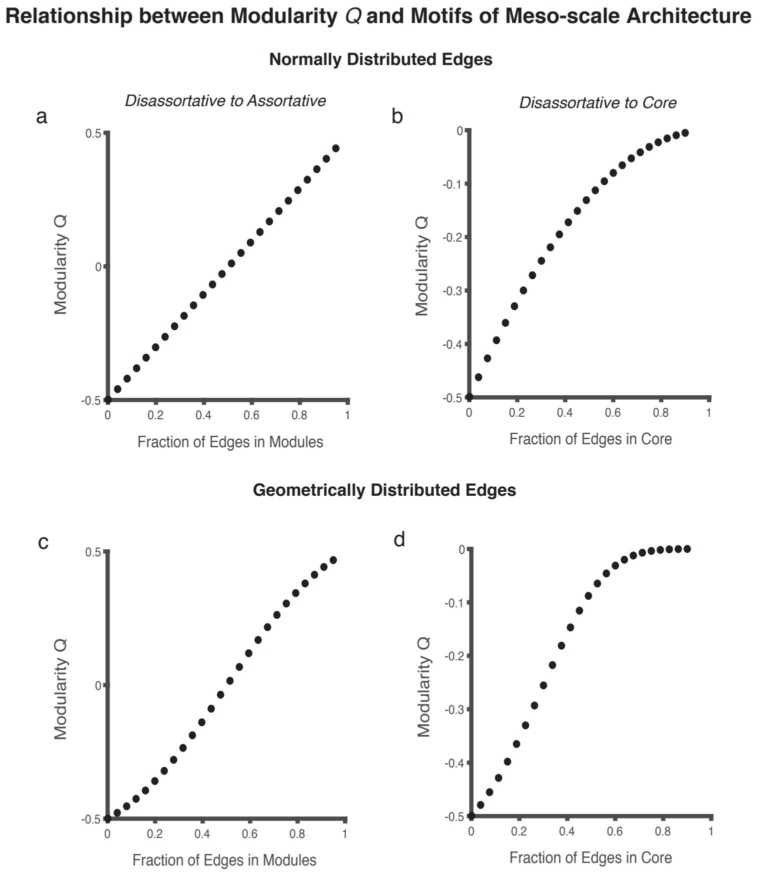

Figure 11: **Relationship between modularity quality Q and mesoscale architecture**. In a network with a 2×2 block structure in the adjacency matrix, we refer to blocks in the $[1, 1]$ and $[2, 2]$ positions as modules. The block in the $[1, 1]$ position is referred to as the core. **(a, c)** As the fraction of total network edges inside of modules increases, that is as the networks becomes more assortative from disassortative, Q increases monotonically. Disassortativity corresponds to a value of $Q = -0.5$, whereas assortativity corresponds to a value of $Q = 0.5$. **(b, d)** Similarly, as the fraction of total network edges inside of the core increases, that is as the networks becomes more core-like from disassortative, Q increases monotonically. Disassortativity corresponds to a value of $Q = -0.5$, whereas a dense core corresponds to a value of $Q = 0$.

References

Danielle S Bassett, Perry Zurn, and Joshua I Gold. On the nature and use of models in network neuroscience. *Nature Reviews Neuroscience*, 19(9):566–578, 2018. ISSN 1471-0048. doi: 10.1038/s41583-018-0038-8. URL https://doi.org/10.1038/s41583-018-0038-8.

Laura Wiles, Shi Gu, Fabio Pasqualetti, Brandon Parvesse, David Gabrieli, Danielle S Bassett, and David F Meaney. Autaptic Connections Shift Network Excitability and Bursting. *Scientific Reports*, 7(1):44006, 2017. ISSN 2045-2322. doi: 10.1038/srep44006. URL https://doi.org/10.1038/srep44006.

Andrew J. Whalen, Sean N. Brennan, Timothy D. Sauer, and Steven J. Schiff. Observability and controllability of nonlinear networks: The role of symmetry. *Phys. Rev. X*, 5:011005, January 2015. doi: 10.1103/PhysRevX.5.011005. URL https://link.aps.org/doi/10.1103/PhysRevX.5.011005.

Sarah Feldt Muldoon, Fabio Pasqualetti, Shi Gu, Matthew Cieslak, Scott T. Grafton, Jean M. Vettel, and Danielle S. Bassett. Stimulation-based control of dynamic brain networks. *PLOS Computational Biology*, 12(9):1–23, 09 2016. doi: 10.1371/journal.pcbi.1005076. URL https://doi.org/10.1371/journal.pcbi.1005076.

Gang Yan, Petra E Vértes, Emma K Towlson, Yee Lian Chew, Denise S Walker, William R Schafer, and Albert-László Barabási. Network control principles predict neuron function in the Caenorhabditis elegans connectome. *Nature*, 550(7677):519–523, 2017. ISSN 1476-4687. doi: 10.1038/nature24056. URL https://doi.org/10.1038/nature24056.

L. Beynel, L. Deng, C.A. Crowell, M. Dannhauer, H. Palmer, S. Hilbig, A.V. Peterchev, B. Luber, S.H. Lisanby, R. Cabeza, L.G. Appelbaum, and S.W. Davis. Structural controllability predicts functional patterns and brain stimulation benefits associated with working memory. *bioRxiv*, 2019. doi: 10.1101/794388. URL https://www.biorxiv.org/content/early/2019/10/18/794388.

James M Shine, Michael Breakspear, Peter T Bell, Kaylena A Ehgoetz Martens, Richard Shine, Oluwasanmi Koyejo, Olaf Sporns, and Russell A Poldrack. Human cognition involves the dynamic integration of neural activity and neuromodulatory systems. *Nature Neuroscience*, 22(2):289–296, 2019. ISSN 1546-1726. doi: 10.1038/s41593-018-0312-0. URL https://doi.org/10.1038/s41593-018-0312-0.

Eli J. Cornblath, Arian Ashourvan, Jason Z. Kim, Richard F. Betzel, Rastko Ciric, Azeez Adebimpe, Graham L. Baum, Xiaosong He, Kosha Ruparel, Tyler M. Moore, Ruben C. Gur, Raquel E. Gur, Russell T. Shinohara, David R. Roalf, Theodore D. Satterthwaite, and Danielle S. Bassett. Temporal sequences of brain activity at rest are constrained by white matter structure and modulated by cognitive demands, 2020.

Zaixu Cui, Jennifer Stiso, Graham L Baum, Jason Z Kim, David R Roalf, Richard F Betzel, Shi Gu, Zhixin Lu, Cedric H Xia, Xiaosong He, Rastko Ciric, Desmond J Oathes, Tyler M Moore, Russell T Shinohara, Kosha Ruparel, Christos Davatzikos, Fabio Pasqualetti, Raquel E Gur, Ruben C Gur, Danielle S Bassett, and Theodore D Satterthwaite. Optimization of energy state transition trajectory supports the development of executive function during youth. *eLife*, 9:e53060, March 2020. ISSN 2050-084X. doi: 10.7554/eLife.53060. URL https://doi.org/10.7554/eLife.53060.

Evelyn Tang, Chad Giusti, Graham L Baum, Shi Gu, Eli Pollock, Ari E Kahn, David R Roalf, Tyler M Moore, Kosha Ruparel, Ruben C Gur, Raquel E Gur, Theodore D Satterthwaite, and Danielle S Bassett. Developmental increases in white matter network controllability support a growing diversity of brain dynamics. *Nature Communications*, 8(1):1252, 2017. ISSN 2041-1723. doi: 10.1038/s41467-017-01254-4. URL https://doi.org/10.1038/s41467-017-01254-4.

Jennifer Stiso, Ankit N. Khambhati, Tommaso Menara, Ari E. Kahn, Joel M. Stein, Sandihitsu R. Das, Richard Gorniak, Joseph Tracy, Brian Litt, Kathryn A. Davis, Fabio Pasqualetti, Timothy H. Lucas, and Danielle S. Bassett. White matter network architecture guides direct electrical stimulation through optimal state transitions. *Cell Reports*, 28(10):2554 – 2566.e7, 2019. ISSN 2211-1247. doi: https://doi.org/10.1016/j.celrep.2019.08.008. URL http://www.sciencedirect.com/science/article/pii/S2211124719310411.

A N Khambhati, A E Kahn, J Costantini, Y Ezzyat, E A Solomon, R E Gross, B C Jobst, S A Sheth, K A Zaghloul, G Worrell, S Seger, B C Lega, S Weiss, M R Sperling, R Gorniak, S R Das, J M Stein, D S Rizzuto, M J Kahana, T H Lucas, K A Davis, J I Tracy, and D S Bassett. Functional control of electrophysiological network architecture using direct neurostimulation in humans. *Netw Neurosci*, 3(3):848–877, 2019.

Yuxiao Yang, Omid G Sani, Edward F Chang, and Maryam M Shanechi. Dynamic network modeling and dimensionality reduction for human ECoG activity. *Journal of Neural Engineering*, 16(5):056014, aug 2019a. ISSN 1741-2552. doi: 10.1088/1741-2552/ab2214. URL https://iopscience.iop.org/article/10.1088/1741-2552/ab2214.

A. Li, S. P. Cornelius, Y.-Y. Liu, L. Wang, and A.-L. Barabási. The fundamental advantages of temporal networks. *Science*, 358(6366):1042–1046, 2017. ISSN 0036-8075. doi: 10.1126/science.aai7488. URL https://science.sciencemag.org/content/358/6366/1042.

Yuxiao Yang, Justin T Lee, Jennifer A Guidera, Ksenia Y Vlasov, JunZhu Pei, Emery N Brown, Ken Solt, and Maryam M Shanechi. Developing a personalized closed-loop controller of medically-induced coma in a rodent model. *Journal of Neural Engineering*, 16(3):036022, apr 2019b. doi: 10.1088/1741-2552/ab0ea4. URL https://doi.org/10.1088%2F1741-2552%2Fab0ea4.

Jorge Gomez Tejeda Zañudo, Gang Yang, and Réka Albert. Structure-based control of complex networks with nonlinear dynamics. *Proceedings of the National Academy of Sciences*, 114(28):7234–7239, 2017. ISSN 0027-8424. doi: 10.1073/pnas.1617387114. URL https://www.pnas.org/content/114/28/7234.
